# Immunological and genetic characterization of women with vulvodynia

**Published:** 2008-11-15

**Authors:** Gerber Stefan, S. Witkin Steven, Stucki David

**Affiliations:** * From Department of Obstetrics and Gynecology , University Hospital Vaud, Lausanne-Switzerland; ** From the Division of Immunology and Infectious Diseases, Department of Obstetrics and Gynecology, Weill Medical College of Cornell University, New York, NY-USA; *** From Obstetrics and Gynecology, State Hospital, 1700 Fribourg - Switzerland

## Abstract

Vulvodynia is a complex disorder and described as discomfort or intense burning pain in the vulvar area. Such chronic pain affects 5 to 15% of women and many suffer of misdiagnosis. For sure the aetiology is multifactorial. Through few studies we consider the inflammatory response plays a major role. There is a genetic profile of women suffering of vulvodynia, especially genetic polymorphisms from genes coding for cytokines, Interleukin-1 receptor antagonist and Interleukin-1beta, and gene coding for mannose-binding lectin (MBL). These polymorphisms result in a stronger inflammatory response and lay these women in a susceptibility situation. Histological analysis showed a chronic no specific inflammation. We have also demonstrated that these patients present in normal state or under infectious induction an inadequate inflammatory response. But there is still a variety of mechanisms which can interact with the inflammatory response. Management of such vulvar pain syndrome could be very frustrating, but the first step for improvement is to get the right diagnosis.

## Introduction 

**Fig. 1:**
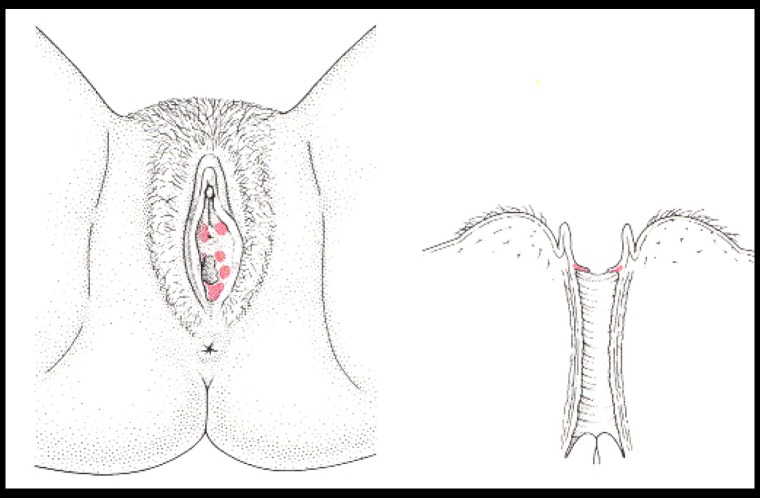
Leibowitch M, Staughton R, Neil S, Barton S, Marwood R. An Atlas of Vulval Disease, a combined dermatological, gynaecological and venereological approach. Second ed 1997, Mosby Dunitz London

Many women experience vulvar pain and discomfort that affects their quality of life. Vulvodynia is a syndrome of vulvar pain and may result for women who experienced such pain in different physical, sexual, social and/or psychological disabilities. Previously termed as „vulvodynia” and „vestibulitis”, vulvar pain syndromes have been reclassified by The International Society for Study of Vulvar Diseases (ISSVD), which established a new classification of vulvodynia [**1**]. Vulvodynia is now classified as generalized or localized depending on the distribution of the pain and also subtyped based on inciting factors; provoked pain or not. The causes of chronic vulvar pain are multifactorial [**2**,**3**]. Out of an infectious aetiology, one of the leading causes of chronic vulvar pain is vestibulodynia. Vestibulodynia represents a type of vulvodynia that is localized only to the vulvar vestibule (**Fig. 1**).

**Fig. 2:**
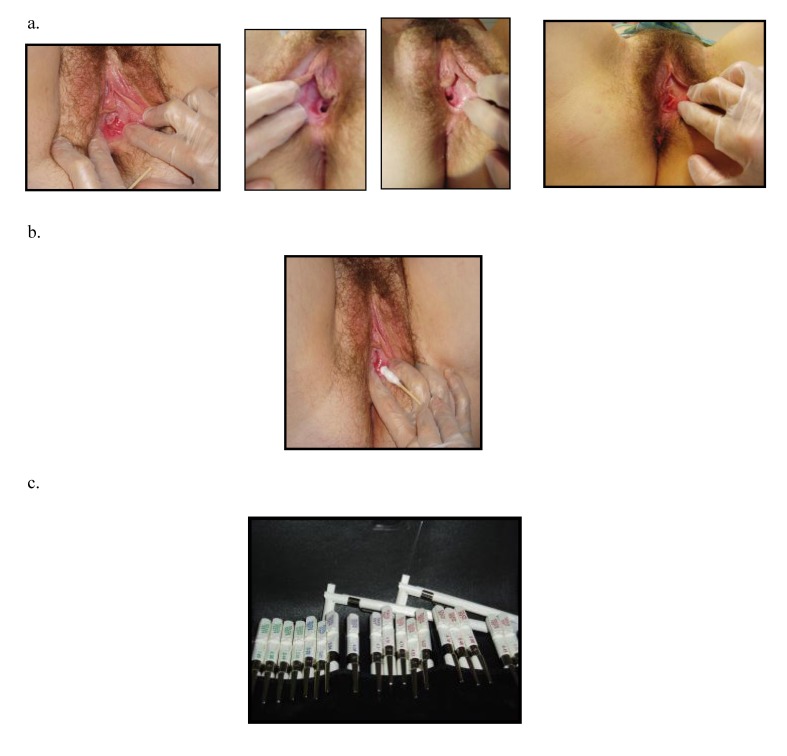
a. Illustration of erythema area in vestibulodynia b. Cotton swab test of painful site c. Quantitative pressure pain threshold assessment through von Frey filament

Vestibulodynia syndrome (VS) is a chronic and painful inflammation of the vestibular structure. Such syndrome was already described at the end of the 19th Century as an excessive sensitivity or an hyperesthesia of the vulvar area [**4**,**5**]. Finally this syndrome was defined as a precise entity by Friedrich in 1987, based only on clinical findings: 1) severe pain on vestibular touch or attempted to vaginal entry, 2) tenderness to pressure localized within the vulvar vestibule (cotton-swab test) and 3) physical findings confined to vestibular erythema [**6**] (**Fig. 2**). However the clinical definition of Friedrich has been subject to varying interpretations, leading to potentially heterogeneous samples in studies [**7**,**8**]. Also there is some doubt about the reliability of the diagnosis of VS. But the study of Bergeron established VS can be reliably diagnosed in women with different degree of vulvodynia and vulvar erythema [**9**].

 The precise incidence of VS in the population is unknown. Although patients are reluctant to express vulvar pain or dyspareunia, the prevalence of VS was estimated as much as 15% in a general gynecological practice [**10**,**11**]. So it’s look like a lot of patients suffer these symptoms, most commonly in young women. The predisposing factors that influence susceptibility to the development of VS have not been identified. Several theories have been proposed but no precise cause of VS has been identified. Consensus accords to a probably multifacorial aetiology [**2**]. Inciting factors have been suggested like environmental or diet factors, neurological dysfunction, allergies, hormonal, iatrogenic or psychological factors [**12**,**13**,**14**]. But vaginal infection has been postulated as a major event that triggers the initiation of clinical symptoms [**13**,**15**,**16**]. Several studies have reported a higher incidence of vulvovaginal candidosis, papillomavirus (HPV) or bacterial vaginosis [**16**-**19**].

Biopsies appear to be of little value in attempt to precise a diagnosis. But they reveal a moderate to severe level of inflammatory infiltrate [**20**,**21**]. Chronic inflammatory cell infiltrate is composed predominantly of T-lymphocytes, plasma cells, mast cells and is involving the mucosal lamina propria and the periglandular connective tissue [**22**,**23**]. Associated to this infiltrate, high level of local cytokines was observed in vulvar and vestibular tissue, suggestive of a hyperimmune response [**24**].

We can hypothesize that women experienced VS can develop an inadequate immune response by a secondary alteration of inflammatory reaction mechanism (cytokines interaction) or by a genetic predisposing factor.

## Genetic characterization of women with Vestibulodynia 

Vestibulodynia syndrome may be the expression of a wider syndrome which aetiology may come from a genetic basis (**Table 1**). In his study, *Goetch* suggested the possibility of a family element in VS since one-third of patients reported having a relative with dyspareunia or vulvodynia [**25**]. In characterization of 162 women with VS, *Witkin* demonstrated a high rate of personal systemic disease, inflammatory bowel disease (22%) and allergy (36%) or a family history of diabetes mellitus (37%) or arthritis (31%) (26). Genetic factor was postulated for women with idiopathic recurrent vulvovaginal candidosis (RVVC) presenting a lower level of Lewis antigen secretor status [**27**,**28**]. 

**Table 1:** Diverse genetic factors in relation to vestibulodynia syndrome

Genetic link	Factor
Hereditary	Family history of dyspareunia
Inflammatory diseases	Asthma, interstitial cystitis, arthritis, Inflammatory bowel disease
Systemic disease	Diabetes, allergy
Ethnic	White and Hispanic women
Genetic polymorphisms	IL-1ra, IL-1β, MBL
IL-1ra: interleukin-1 receptor antagonist, IL-1 β: interleukin-1 β, MBL: mannose-binding lectin	

This genetic susceptibility may play a role in the development of VS according to several observations of higher RVVC in women with VS than control patients. Additionally RVVC or frequent use of local medication may result in the development of cross-reacting immunological response to self antigens in some women, inducing autoimmune reaction and chronic inflammation [**29**].

A higher incidence of VS in white women can illustrate a genetic predisposition [**30**]. This observation can be related to an ethnic distribution of a specific genotype. The gene coding for interleukin-1 receptor antagonist (IL-1RA), an anti-inflammatory cytokine, is polymorphic in human subjects [**31**]. The presence of allele 2 of IL-1RA gene (IL-1RN*2) has been associated with increase interleukin-1β (IL-1β) activity, pro-inflammatory cytokine, resulting in a prolonged and increased inflammatory response [**32**,**33**]. Homozygosity for allele 2 has been shown to be more prevalent in women with VS than in control women, respectively 53% and 9% [**34**]. Such genotype also is more prevalent in white population [**35**,**36**]. Women with VVS, who are homozygous for IL-1RN*2 present different characteristics compare to the other genotypes [**26**]. They have a younger age of symptom onset, a stronger association with history of allergy and a greater frequency of HPV in the vaginal vestibule. But, surprisingly, there was no relation between the intensity of vestibular pain and IL-1RA genotype. So, this homozygosity for allele 2 of IL-1RA gene appears as a genetic deficiency in the ability to terminate the inflammatory response and allows the development to a chronic inflammation. 

Taking into consideration the inflammatory induction side, studies has been performed about IL-1β. The gene coding for IL-1β is polymorphic too [**37**,**38**]. Allele 2 at the +3953 locus has been associated to a higher production of IL-1β. In our study of 59 women with VS, we observed a greater frequency of allele 2 in VS patients than in control group, respectively 46% and 25% [**39**]. As expected in both groups, possession of allele 2 of this polymorphic gene resulted in a small increase in induced IL-1β production. Although the genes for IL-1RA and IL-1β are both located on the same chromosome 2, we did not observe any relationship between the different alleles. Perhaps other polymorphic loci will show a direct relationship influencing susceptibility to VS. Or the both genes could stay as independent inducing factor.

## Immunological characterization of women with Vestibulodynia

Biopsies of affected regions of the vestibule of women with VS show a chronic inflammatory infiltrate [**20**,**21**] (**Table 2**). In the assessment of pro-inflammatory cytokines in these tissues, median levels of IL-1β and tumor necrosis factor-α (TNF-α) were elevated 2.3 fold and 1.8 fold, respectively, in VS patients compare to asymptomatic patients [**24**]. Although level can vary according to anatomic site, this local inflammatory cytokines elevation may contribute to the pathophysiology mechanism of hyperalgesia. Interleukin-1 receptor antagonist is a natural competitor of IL-1β and is normally present in the circulation [**32**,**33**,**40**]. Interesting to observe the level of IL-1ra is much higher in asymptomatic women compared to women with VS [**41**].These women appear to be low producers of IL-1ra, which can induce and/or install the development of a chronic inflammatory reaction.

**Table 2:** Immunological factors related to vestibulodynia

Immunological field	Factor
Histology	T-lymphocyte infiltrate, plasma cells Mast cells
Neurogenic inflammation	NGF, CGRP, SP
Steroid inflammation	Steroid receptor
Cytokines production	IL-1ra, IL-1 β, TNF-α, IFN-α
NGF: nerve growth factor , CGRP: calcitonin gene-related peptide SP: substance p IL-1ra: interleukin-1 receptor antagonist, IL-1 β: interleukin-1 β, TNFα: tumor necrosis factor alpha, IFNα: interferon alpha	

In a previous study we compared pro- and anti-inflammatory immune response after two different ways of stimulation, 70kDa heat shock protein (HSP70) and lipopolysaccharide, in whole blood culture from 62 women with VS and 48 controls [**41**]. Expression of the stress response protein, HSP70, is up regulated under non-physilogical conditions and induces pro-inflammatory cytokines [**42**]. In induced blood with HSP70, median levels of IL-1β were higher in VS patients and, conversely, levels of IL-1ra were higher in control patients. In response to lipopolysaccharide induction, median levels of IL-1β were similar in both groups but controls produced a higher concentration of IL-1ra. Interesting to note, considering the two kind of models of stimulation, the ratio IL-1ra to IL-1β was higher in control patients. Theses results demonstrate the relative inability to down-regulate pro-inflammatory activity in women with VS. Other polymorphisms in gene linking with an inflammatory process were identified, like mannose-binding lectin [**43**].

Previous studies have suggested a possible benefit of local injection of interferon-α (IFN-α) [**44**,**45**]. Interferon-α is a potent activator of natural killer cells and acts as an inducer of IL-1ra [**46**]. In uninduced and induced whole blood culture, IFN-α was present in a higher rate in controls, 68% and 70%, than VS patients, respectively, 34% and 48% [**47**]. But among the only positive samples of VS patients, there was no difference in the levels of IFN-α in this group and controls! There was no relation between IL-1ra genotype and IFN-α production. Interferon-α production under the same conditions was similar in controls and VS patients. Such observation seems demonstrated there is an other identified group of women with VS who are unable to produce specifically IFN-α.

## Conclusions

The present review clearly demonstrates that women with VS differ from other women in the immune response. Cytokines production plays a major role in the development of the syndrome and mediates the progression of a local inflammation associated to a hyperlagesia and chronic pain. Looking on the balance between pro- and anti-inflammatory cytokines production, under any kind of stimulation, women with VS always present a final ratio in favour to pro-inflammatory response. 

**Table 3:**
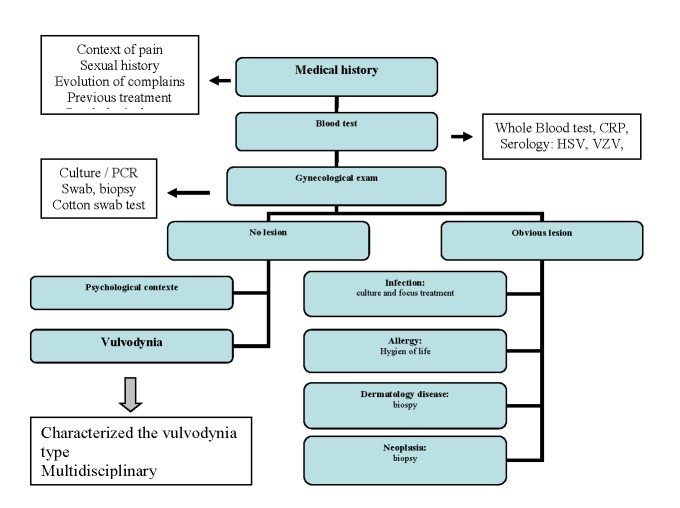
Vulvodynia screening algorithm. All steps must be performed

Complexity of cytokines interactions and regulatory mechanisms can explain how is difficult to find precisely the level of dysfunction. Also the polymorphism of cytokine genes has in diverse situation a direct effect on the cytokines production. The final result of such „dysfunction” of the immune response is to place these women in a high vulnerability, in which they look unable to down-regulate the pro-inflammatory cytokines cascade. Regardless of the type of trigger agent (infectious, toxic chemical, mechanic or allergic), which could be just transitory, the induction-inflammatory event result in persistence of chronic inflammation, also after clearance of the trigger agent.

But majors question are still open. Why unbalanced immune response is located in a horseshoe-shaped area of the lower vestibule? In the future, new studies must be involved in research of local factors, as specific cytokines cellular receptor (s) or topical modulator (s) of cytokines expression. Until we can clarify the aetiology of this disease the first major step must be from our part to get the right diagnosis. Vulvodynia is a diagnosis of exclusion, a pain syndrome with no other identified cause. We must keep in mind an adequate algorithm to identify these patients [**48**,**49**] (**Table 3**). Giving the right diagnosis is already a relief for such patients. There is no consensus regarding the appropriate treatment and most of available evidence comes from clinical experience [**2**,**14**,**48**,**50**,**51**]. According to a bad quality of life, women suffering of vulvodynia need a multidisciplinary management, including emotional and psychological support.
